# A Critical Juncture in Fiscal Federalism? Canada's Response to COVID-19

**DOI:** 10.1017/S0008423920000323

**Published:** 2020-04-20

**Authors:** Daniel Béland, André Lecours, Mireille Paquet, Trevor Tombe

**Affiliations:** 1 Department of Political Science, McGill University, Montreal, QC, H3A 0G2; 2 School of Political Studies, University of Ottawa, Ottawa, ON, K1N 6N5; 3 Department of Political Science, Concordia University, Montreal, QC, H3 G 1M8; 4 Department of Economics, University of Calgary, Calgary, AB, T2N 1N4

## Abstract

The COVID-19 crisis could trigger a critical juncture for several institutional arrangements in Canada, potentially leading to notable changes in fiscal federalism. This research note combines insights from historical institutionalism with recent economic and fiscal projections to explore avenues for reform in response to the COVID-19 crisis. Given the magnitude of the crisis, provincial governments may be unable to absorb the fiscal costs on their own. But vast differences in fiscal and economic circumstances across provinces make federal arrangements difficult to design. We argue that intergovernmental power dynamics and the principle of provincial autonomy are particularly important considerations in thinking about fiscal federalism post–COVID-19.

The COVID-19 crisis could trigger a critical juncture for several institutional arrangements in Canada, potentially leading to notable changes in fiscal federalism. This research note combines insights from historical institutionalism with recent economic and fiscal projections to explore avenues for reform in response to the COVID-19 crisis. Given the magnitude of the crisis, provincial governments may be unable to absorb the fiscal costs on their own. But vast differences in fiscal and economic circumstances across provinces make federal arrangements difficult to design. We argue that intergovernmental power dynamics and the principle of provincial autonomy are particularly important considerations in thinking about fiscal federalism post–COVID-19.

## Critical Junctures and Federalism

Much has been written about the inertia and stickiness of public policies, which can become increasingly entrenched over time through self-reinforcing feedback mechanisms leading to path dependence (Pierson, 2000). There is strong evidence that institutional continuity is a central aspect of policy development, both during and between crises (Campbell, 2004). Yet recent literature on policy change inspired by historical institutionalism also stresses the existence of self-undermining mechanisms that can lead to the gradual erosion of existing policies (Jacobs and Weaver, 2015). This emphasis on self-undermining mechanisms has added to previous insights about exogenous shocks as a trigger for institutional and policy change (Pierson, 2000).

Central to historical institutionalism is the idea of institutions as regimes (Mahoney and Thelen, 2010): the dynamic “relationships between actors [which], through formal and informal rules, organize the distribution of resources and power” (Paquet, 2019: 21). The Canadian federation represents such a regime where, in addition to the formal division of responsibilities, power and resources are distributed dynamically through interactions between federal and provincial governments. In this context, policy feedback is as much about the costs of deviating from existing policy solutions as it is about the behaviour of institutional actors, which reflects both their core principles and their actual capacities. In Canada, nowhere is this more visible than in fiscal federalism. As Keith Banting (1987) and Alain Noël (2008)—among others—have amply discussed, the fiscal arrangements that distribute resources within the federation illustrate some measure of agreement over core principles that shape the behaviour of governments.

While they can transform or drift over time, abrupt changes in institutional regimes typically follow large-scale crises such as economic depressions, energy shocks, global pandemics and world wars (Campbell, 2004: 174). Institutionalist scholars understand such crises as “critical junctures” during which political actors have much more capacity than usual to fight policy inertia and bring about transformative change. As James Mahoney (2002: 7) argues, “critical junctures are moments of relative structural indeterminism when willful actors shape outcomes in a more voluntaristic fashion than normal circumstances permit.” The development of many important features of Canada's current arrangements, for example, emerged out of the Great Depression and World War II. And out of these critical junctures emerged a new consensus over the principles in which redistribution should be embedded (Jenson, 1997), subject to the time-specific interests and capacities of the federal and provincial governments.

The COVID-19 crisis may be another critical juncture that opens the door to new approaches to Canadian fiscal federalism. As historical institutionalism has taught us, these alternatives are much more than technical debates; they both emerge from and shape collective understandings of federalism and power relations among governments.

## Fiscal Federalism in Canada

Federal transfers to provincial governments are central to fiscal federalism in Canada. There are three major transfers: the Canada Health Transfer (CHT), a per capita transfer meant to support provincial healthcare systems; the Canada Social Transfer (CST), also a per capita transfer intended to support post-secondary education, social assistance and child services; and equalization, an unconditional transfer to provinces with fiscal capacity below a national standard, whose principle is enshrined in the 1982 Constitution Act (Béland et al., 2017). The CHT represents approximately 47 per cent of the major transfers; equalization about 25 per cent; and CST approximately 20 per cent. Transfers embody core political principles such as substantive equality among both citizens and governments, provincial autonomy, and economic and fiscal sustainability.

Prior to COVID-19, fiscal arrangements were increasingly challenged by provinces. These disputes represent important legacies that could shape how fiscal federalism responds to COVID-19. Some of these pressures had strong political expressions. The government of Alberta made it clear that it felt unfairly treated in the federation. Resistance by the federal government and some other provinces to certain pipeline projects, in the context of Alberta's weak economic performance, provoked such discontent that the provincial government established the Fair Deal Panel to consult Albertans about their place in the federation. A key theme here was that the province contributes more to the country than it receives. This assessment triggered forceful criticism of equalization, with Alberta Premier Jason Kenney promising to hold a referendum on equalization if he found progress on pipeline expansion to be unsatisfactory. Because no change to equalization would make Alberta a recipient, the province also pressured Ottawa to reform its Fiscal Stabilization Program so Alberta could be compensated for swift downturns in its economy (Tombe, 2020). The current crisis, which is hurting the whole country, could dampen Alberta's grievances.

Beyond these public rows involving Alberta, fiscal federalism in Canada was also experiencing, prior to the COVID-19 crisis, significant structural challenges that were of concern to many provinces. Population aging and out-migration have placed some provinces, particularly Newfoundland and Labrador, in a precarious fiscal situation. Federal healthcare financing has been blind to the specific situations of provinces, a situation lamented by Quebec and British Columbia, among others. The CST is similarly unresponsive to specific provincial needs. From an intergovernmental perspective, bilateral agreements around federal transfers for infrastructure and housing have been difficult to negotiate, especially with the Quebec government.

## Options for Reform

The Canadian response to COVID-19 and reforms to fiscal transfer arrangements will be shaped by existing policy legacies as much as by current power relations in the federation. Respecting provincial autonomy will likely remain a key political and policy consideration, although the federal government's dominant fiscal capacity, a considerable source of federal power, could be deployed at a time when the provinces badly need it. But in the short term, the speed of the COVID-19 crisis necessitates working within existing arrangements. Two programs stand out.

First, to address provincial spending pressures, the federal government can use its existing disaster assistance program with only minor modification. The federal Disaster Financial Assistance Arrangements (DFAA) aims to “assist provinces with the costs of dealing with a disaster where those costs would otherwise place a significant burden on the provincial economy and would exceed what they might reasonably be expected to fully bear on their own” (Government of Canada, 2020). COVID-19 is clearly such an event. Yet the DFAA explicitly excludes “pandemic health emergencies,” though the restriction is regulatory in nature. Cabinet could therefore change the DFAA guidelines and insulate provinces from many of the direct COVID-19 costs under the current formula. For perspective, a Canadian package equivalent to the United States’ $150 billion (US) Coronavirus Relief Fund—which will aid state and local governments—would be $630 per person to provinces at a cost of roughly $23 billion (CAN).

Second, to address provincial revenue pressures, the federal government may expand the Fiscal Stabilization Program. Given the scale of the economic contraction, provincial own-source revenues could feasibly decline by 10 per cent—or around $40 billion—although much uncertainty remains. The current Fiscal Stabilization Program will cover only a small portion of these losses, as total payments are limited to $60 per capita for a total of just over $2 billion nationally. Existing legislation, however, provides the minister of finance discretion to provide interest-free loans for a period of five years—buying time for a more comprehensive solution. Going forward, the COVID-19 shock may strengthen the case some provinces have made—most recently Alberta, as previously discussed—for a dramatically expanded and potentially uncapped program.

Both short-term measures respect provincial autonomy, but as the expediency of the moment wanes, deeper changes may be on the table. There is no shortage of fiscal pressure, including the dire financial situation of various municipalities (Mason, 2020), but pre-existing challenges in healthcare financing may lead it to the top of the national agenda.

The CHT could be enlarged and adjusted to increase funding more quickly to provinces with more challenging demographic pressures—a long-standing provincial demand by some, notably Quebec, although one opposed by others, such as Alberta. Regardless, an immediate boost of $6 billion would grow CHT to one-quarter of provincial health spending (a level not seen since the late 1970s). If implemented as an “age-adjustment” to the current transfer, it could provide a larger benefit to provinces with comparatively older populations (especially the Atlantic provinces). This could not only strengthen health systems, in general, but better prepare for future health crises because, as we have seen with COVID-19, elderly populations are more vulnerable. The federal government may also consider returning to funding assistance in other areas, notably education—particularly post-secondary—hit hard by the COVID-19 crisis.

As federal fiscal capacity and sustainability vastly exceed those of the provinces, even more dramatic re-evaluations of fiscal arrangements are possible. For example, following the Great Depression, the Rowell-Sirois Commission recommended that the federal government take on provincial debt (RCDPR, 1940). There may be renewed pressure to consider this option, at least in part. Newfoundland and Labrador, which faces both COVID-19 and low oil prices, now relies on Bank of Canada purchases of provincial debt. Though important to ensure market liquidity, this debt remains on provincial balance sheets and may strain fiscal sustainability. The federal government could step in. In the extreme, shifting the total provincial net financial liabilities of roughly $700 billion to the federal government would roughly double Canada's net debt to gross domestic product (GDP) position from its current 30 per cent to nearly 60 per cent: a large increase, but just marginally above its 1999 level—and significantly below that of the United States. And given today's low rates, the higher federal interest costs are equivalent to barely over one percentage point of the goods and services tax (GST). This is not to say such a move is wise, only that federal fiscal capacity is difficult to overstate.

Regardless of how fiscal federalism responds or what the specific design details are, the COVID-19 crisis potentially represents a critical juncture with lasting implications for fiscal federalism in Canada.
